# Preventive Oral-Health Standardization, Program-Evaluation and Replication Toolkit (PROSPER-OH): A Standardized, Non-clinical Toolkit for Adapting, Implementing, and Evaluating Community Preventive Oral-Health Programs in Underserved Settings

**DOI:** 10.7759/cureus.113738

**Published:** 2026-07-31

**Authors:** Ariadine Olimpio da Paz

**Affiliations:** 1 Dentistry, Independent Research, Orlando, USA

**Keywords:** community health, consolidated framework for implementation research, health equity, implementation science, measurement, oral health, preventive dentistry, program evaluation, re-aim, underserved populations

## Abstract

Community preventive oral-health programs (collective and topical fluoride, supervised toothbrushing, school-based dental sealant programs, and community education) have an established evidence base for caries prevention, yet their adaptation, delivery fidelity, and measurement are rarely standardized across sites. This limits comparability, replicability, and equity accountability, especially in underserved settings where oral-disease burden is concentrated. This report describes Preventive Oral-health Standardization, Program-Evaluation and Replication Toolkit (PROSPER-OH), an open, non-clinical toolkit that standardizes how a community preventive oral-health program is assessed for context readiness, adapted, delivered with fidelity, and, above all, measured in a replicable, equity-stratified way. PROSPER-OH operationalizes two established implementation - science frameworks, the Consolidated Framework for Implementation Research (CFIR; context and readiness) and Reach, Effectiveness, Adoption, Implementation, Maintenance (RE-AIM; the measurement and continuity layers), into five core modules, two extension modules, and two non-core add-ons. Its distinctive component is a standardized Core Indicator Set of 24 program-level indicators with mandatory equity stratification (geography, socioeconomic status, age band, and language need) and a trilingual Portuguese/Spanish/English codebook, combined with a 0-100 Program Quality Scorecard that places a program on a four-level maturity ladder and aligns reporting fields to federal program metrics (Health Resources and Services Administration, Centers for Disease Control and Prevention, Medicaid/Early and Periodic Screening, Diagnostic and Treatment, and Healthy People 2030). Every instrument is de-identified and aggregated by design: the toolkit operates on programs and populations, never on individual patients, and performs no diagnosis, screening, individual-risk determination, or clinical act. An illustrative, explicitly synthetic state-level walkthrough demonstrates how the modules chain together in an officially designated underserved context. PROSPER-OH provides an author-independent measurement and implementation architecture designed to support replication across settings. It is released openly for replication and feedback and has not yet been empirically validated.

## Introduction

Oral diseases affect an estimated 3.7 billion people worldwide, and untreated dental caries in permanent teeth is the single most common health condition globally, with the burden falling disproportionately on low-income, disadvantaged, and underserved populations [1,2]. Dental caries is also the most common chronic disease of children and adults in the United States. Approximately 23% of children aged two to five years had caries in their primary teeth in 2011-2016, and untreated tooth decay was estimated to cost US$45.9 billion in lost productivity in 2015 [3,4]. This burden is concentrated in officially designated underserved areas. As of March 31, 2026, the Health Resources and Services Administration (HRSA) recorded 7,702 Dental Health Professional Shortage Area (HPSA) designations covering 70,612,204 people, with only 33.51% of need met and an estimated 11,779 additional dentists required to remove the designations [5].

Where preventive programs and a working referral pathway are absent, populations fall back on hospital emergency departments: an estimated 2.1 million visits for dental conditions per year, at a total cost of about US$2.7 billion [6]. These are largely preventable, non-definitive encounters that a community preventive program is designed to displace. Public coverage exists but is unevenly used: dental care is a required Early and Periodic Screening, Diagnostic, and Treatment (EPSDT) benefit for children enrolled in Medicaid, yet in 2018 only 41.5% of Medicaid- and Children's Health Insurance Program-enrolled children received an oral examination and 47.1% received a topical fluoride treatment [7]. National targets frame the gap: Healthy People 2030 set out to reduce untreated decay in children and adolescents from 16.0% (2013-2016) to 12.5% (objective OH-02) and to increase use of the oral-health care system from a 2016 baseline of 43.2% to 45.0% (objective OH-08); the latter target has since been met, reaching 45.5% in 2022, an encouraging trend that standardized program measurement can help sustain and extend [8].

Community preventive interventions (collective and topical fluoride, supervised toothbrushing, and school-based dental sealant programs) are supported by an established evidence base for caries prevention and are recommended for population-level delivery [9-13]. These interventions are also central to reducing oral-health inequalities, since dental caries follows a socially patterned distribution linked to shared upstream determinants such as income, education, and access to care, and community-level delivery is itself a recognized strategy for narrowing that gradient [14]. What is frequently missing is not the intervention itself but a standardized way to adapt, deliver, and, above all, measure such programs comparably across sites. Programs delivered by different teams, in different settings, and in different languages are difficult to compare, reproduce, or hold accountable for equitable reach when each site improvises its own metrics.

This report describes the Preventive Oral-health Standardization, Program-Evaluation and Replication Toolkit (PROSPER-OH), which addresses this gap by operationalizing two established implementation science frameworks - Consolidated Framework for Implementation Research (CFIR), for assessing context and readiness [15,16], and Reach, Effectiveness, Adoption, Implementation, Maintenance (RE-AIM), for the indicator set, fidelity, scorecard, training, and evaluation components [17] - into a reusable, non-clinical measurement and implementation architecture. PROSPER-OH is presented here as a proposed framework that has not yet undergone empirical validation. The objective is to provide an open toolkit that enables any local public health team to adapt, implement, and evaluate community preventive oral-health programs in underserved settings in a replicable, comparable, and equity-stratified way, without depending on the original developer. Existing resources address parts of this gap without fully closing it: general program-evaluation guidance, such as the Association of State and Territorial Dental Directors (ASTDD) Best Practice Approaches and the Centers for Disease Control and Prevention (CDC) Program Evaluation Framework, offers process guidance but not a fixed, cross-site-comparable indicator set or maturity score, while many community oral-health program evaluations rely on individual-level clinical indices (e.g., Decayed, Missing, and Filled Teeth (DMFT) index and gingival index) collected through clinical examination, a scope PROSPER-OH deliberately excludes by design [9,18].

## Technical report

Design principles

PROSPER-OH is built on four design principles. First, it adapts, and does not originate, established third-party frameworks: CFIR for the context/readiness assessment and RE-AIM for the reach, adoption, implementation, and maintenance layers, together with the well-described separation of an intervention's “core components” from its “adaptable periphery” [15-17], and its indicator, scorecard, and replication-report design draws on established public health program-evaluation methodology [18]. Second, it is author-independent by construction: every instrument, codebook, and reporting template is specified in enough detail that a team which has never worked with the developer can reproduce a program at a new site. Third, equity is built in rather than added on: disaggregation by geography, socioeconomic status, age band, and language need is mandatory, and a trilingual Portuguese/Spanish/English codebook is provided to support consistent service to Lusophone and Hispanic underserved populations, pending formal cross-cultural validation. Fourth, and most importantly, the toolkit maintains a strict non-clinical scope: it operates on programs and populations, never on individual patients, and performs no diagnosis, screening, individual-risk determination, or clinical act. Every instrument is de-identified and aggregate.

Architecture

The toolkit comprises seven numbered modules and two non-core add-ons. The core package (M1, M3, M5, M6, M7) is a coherent, self-contained toolkit on its own; the extension modules (M2, M4) and non-core add-ons (D1, D2) are additive. Table 1 summarizes the architecture.

**Table 1:** PROSPER-OH module architecture M1-M7: Module 1 through Module 7 (core toolkit components); D1-D2: Non-core add-ons 1-2; CFIR: Consolidated Framework for Implementation Research; PROSPER-OH: Preventive Oral-health Standardization, Program-Evaluation and Replication Toolkit

Module	Title	Layer
M1	Context Readiness and CFIR-adapted Assessment	Core
M2	Intervention Adaptation Matrix (core function vs. adaptable form)	Extension
M3	Core Indicator Set, data-collection instruments, and equity stratification	Core
M4	Fidelity and Adoption Checklist	Extension
M5	Capacity Tiers and Program Quality Scorecard (0-100)	Core
M6	Knowledge-Transfer and Implementation Training (non-clinical)	Core
M7	Evaluation and Replication Logbook and grant-aligned reporting template	Core
D1	Referral-Pathway Readiness (program-level)	Non-core
D2	Elderly-Stratum Disaggregation add-on	Non-core

Context readiness and intervention adaptation (M1, M2)

M1 is the entry-point readiness layer. It adapts CFIR into a short, reusable, program-level assessment that reads the setting a program will run in: the outer setting (underserved designation, area-level burden, access barriers, partner sectors), the inner setting (unit resources, multiprofessional team, data infrastructure), the individuals-as-implementers (staff capacity, explicitly reframed as the team rather than patients), and the process of standing the program up. Every item is answered from a document, a roster, or an official area designation; the instrument contains no person-level item, and its output is a context-readiness profile by CFIR domain rather than any clinical judgment.

M2 is an intervention adaptation matrix that separates, for each program, the core function (parameters that must not change for the program to retain its population effect) from the adaptable form (surface features a program or guideline permits to vary by setting, language, or age band). It is a documentation instrument: it records where each parameter is specified (a public program, a published guideline, or planned systematic-review evidence) and never determines a clinical parameter itself. When an adaptation would force a change to a core parameter, that is recorded as a change of program rather than an adaptation, providing an explicit fidelity boundary.

The Core Indicator Set and equity stratification (M3)

M3 is the standardized measurement layer and the toolkit's distinctive contribution. It defines a compact library of 24 program-level indicators, organized by RE-AIM domain, that any team can adopt to plan, monitor, and compare a preventive program across sites. Each indicator is specified with six fields: stable identifier, name, numerator/denominator (population/program counts only), periodicity, aggregate source, and the equity stratifiers that must be reported. No indicator uses an individual clinical outcome. Table 2 summarizes the indicator families.

**Table 2:** Core Indicator Set: families and coverage (24 indicators) RE-AIM: Reach, Effectiveness, Adoption, Implementation, Maintenance

RE-AIM domain	Count	What it captures (program-level)
Reach	6	Coverage and participation across school-age, collective fluoride, supervised toothbrushing, education, community events, and vulnerable groups
Effectiveness	4	Program-level signals: education completion, continuity, aggregate self-reported uptake, referral-pathway completion (not individual clinical endpoints)
Adoption	3	Uptake by sites, staff, and partner sectors
Implementation	4	Session-delivery fidelity, documentation completeness, authorization compliance, data-collection completeness
Maintenance	4	Program persistence, re-coverage, trained-team retention, evaluation cadence
Equity	3	Rural coverage gap, low-income reach, language-access delivery

Every Reach and Effectiveness indicator must be reported disaggregated by, at minimum, four stratifiers: geography (rural/urban and HPSA/Medically Underserved Area flag where applicable), socioeconomic stratum, age band (child/adolescent/adult/elderly), and language need. Reporting a headline number without these stratifiers is defined as a fidelity failure and is itself flagged by an implementation indicator. A trilingual Portuguese/Spanish/English codebook accompanies each indicator to support consistent collection across language settings, it was translated using the author’s native fluency in Portuguese and childhood fluency in Spanish, and has not undergone formal cross-cultural validation (e.g., back-translation, external review, or cognitive testing), which is planned as future work, and all data-collection instruments (an aggregate session log, a coverage worksheet, an anonymous program survey, and a referral-pathway log) are de-identified and aggregate by design.

Fidelity, maturity scoring, training, and continuity (M4-M7)

M4 is a program-level fidelity-and-adoption checklist that verifies whether a program cycle was delivered on schedule, recorded, staffed by a trained team, authorized by the partner site, and incorporated into routine: capturing the administrative footprint of a cycle from documents, never the clinical correctness of any act on a person.

M5 turns the indicators and operational state of a program into a single comparable 0-100 Program Quality Scorecard and places the program on a four-level maturity ladder. The score is the sum of five weighted dimensions (Table 3), and the score band determines the tier (Table 4). Weights emphasize measurement because standardized measurement is the toolkit's distinctive contribution and the hook for adoption without an outside expert.

**Table 3:** Program Quality Scorecard dimensions and weights (0-100) Provisional, pending pilot and sensitivity testing.

Dimension	Weight	What it measures (program-level)
A. Delivery	20	The program is delivered to defined target groups
B. Measurement	25	The Core Indicator Set is collected with required equity stratification
C. Fidelity and governance	20	Sessions follow the M4 checklist; authorizations and documentation are on file
D. Workforce capacity	15	The local team is trained and retained to operate the program and collect data
E. Evaluation and replication	20	A recurring evaluation cycle runs and a replication-ready report exists

**Table 4:** Score bands and capacity tiers Provisional, pending pilot and sensitivity testing.

Score	Tier
0-24	Tier 0: No standardized program
25-54	Tier 1: Established delivery
55-79	Tier 2: Measured program
80-100	Tier 3: Continuously evaluated and replicable

M6 is the non-clinical train-the-local-team package: a curriculum, a facilitator script, and reusable materials so a team can operate the program and collect the data without any single outside expert present. It is the highest-care boundary module and therefore states explicitly what it does not train: it does not train anyone to apply fluoride, brush another person's teeth, perform any procedure, diagnose, screen, treat, or prescribe. What it trains is the operation of a population program: delivering preventive education, producing education materials, collecting the M3 indicators, applying the M4 checklist, and reading an M5/M7 evaluation.

M7 is the continuity layer. It provides an evaluation logbook (one row per cycle recording adaptations, fidelity, indicators reported, and lessons learned), a standardized replication report that lets another site reproduce a program without the original developer, and a grant-aligned reporting template whose fields line up with HRSA, CDC, and Medicaid program metrics, plus a clearly labeled avoided-cost estimate. The replication report is the operational vehicle for the toolkit's intended author-independent replicability, which remains to be demonstrated through multi-site implementation.

Non-core add-ons (D1, D2)

D1 is a program-level referral-pathway readiness instrument. It answers one question only: does the program have an approved, documented referral workflow and a trained non-dental team to operate it, read entirely from documents. It is explicitly not a screening, risk-flagging, triage, or case-finding tool, and contains zero individual signs, symptoms, risk categories, or findings. D2 is a stratification lens, not a new module: it shows how the existing modules already disaggregate results by an elderly population stratum through the age-band equity stratifier, adding no new indicator or data field.

Illustrative application

To demonstrate how the modules chain together, the toolkit includes an explicitly synthetic, state-level walkthrough anchored in an officially designated underserved context, in which “underserved” is read from a federal HPSA designation rather than any local screening (Table 5). The walkthrough runs M1 (read context readiness) to M3 (collect the equity-stratified Core Indicator Set with the trilingual codebook) to M5 (score program maturity 0-100 and assign a tier) to M7 (record the cycle, produce an author-independent replication report, and populate the grant-aligned template). It is a planning illustration only: no program is described as running, every figure is program- or population-level and aggregate, and the same chain applies unchanged to any state with official designations. State-level counterparts to the national figures are treated as placeholders to be verified from the same federal sources.

**Table 5:** Core module chain (M1 to M7) Core chain from the illustrative application walkthrough: M1 (context readiness) precedes data collection in M3 (24-indicator Core Indicator Set); M3 results are aggregated into the M5 Program Quality Scorecard; M5 findings are recorded and used to produce the M7 replication report. Extension modules M2 (intervention adaptation) and M4 (fidelity and adoption checklist) feed into the M1-M3 and M3-M5 transitions, respectively; M6 (knowledge-transfer training) prepares the team that operates the full chain. M7 findings feed the next M1 reassessment, making the chain iterative across cycles. CFIR: Consolidated Framework for Implementation Research

M1	→	M3	→	M5	→	M7
Context readiness (CFIR)		24 core indicators		0-100 scorecard, four tiers		Evaluation log and replication report

Non-clinical scope and safeguards

The non-clinical boundary is enforced structurally rather than by disclaimer alone. The unit of analysis in every instrument is the program, the population, or the setting; no instrument contains a person-level field; every affirmative answer in a checklist is backed by a document rather than by observing a person; and each module states explicitly what it does not do. Clinical need is routed to licensed services through the program-level referral-pathway readiness of D1, which the team is trained to operate but not to provide.

## Discussion

PROSPER-OH's distinctive element is not a new intervention but a standardized measurement and implementation layer wrapped around interventions that already have an evidence base. Three features differentiate it from ad hoc program monitoring. First, measurement is standardized and comparable: a fixed 24-indicator Core Indicator Set with a single scoring rubric lets programs be compared across sites and over time, and lets a program's maturity be expressed as one number and one tier. Second, equity accountability is mandatory rather than optional, because disaggregation by geography, socioeconomic status, age band, and language need is built into the definition of correct reporting, and the trilingual codebook operationalizes language access. Third, replicability is author-independent by design: the M7 replication report is constructed so that a team elsewhere can reproduce a program without the developer. This design facilitates replication but does not itself demonstrate it; establishing actual feasibility, reliability, and replicability requires multi-site implementation studies, as discussed under Limitations below. Alignment of reporting fields to federal program metrics (HRSA, CDC, Medicaid/EPSDT, and Healthy People 2030) is intended to lower the reporting burden for sites that pursue public funding, without any claim that a program is funded or fundable.

The toolkit sits within, and depends on, established work. It adapts CFIR and RE-AIM and the core-component/adaptable-periphery distinction rather than proposing a new framework [15-17], and it is consistent with best-practice guidance for state and community oral-health programs [9]. Its contribution is domain-specific operationalization: turning those general frameworks into a concrete, non-clinical, equity-stratified toolkit for community preventive oral-health programs in underserved settings. The complete toolkit, including all module templates and the trilingual codebook, is openly deposited for replication and reuse [19].

Limitations

Several limitations should be stated plainly. The toolkit has not been empirically validated, adopted, or placed in routine use; this report describes an artifact and its design rationale, not results from deployment. The indicators are process, coverage, and program-maturity measures, not individual clinical outcomes, so the toolkit measures how well a program is delivered and measured, not whether a population's caries experience changed; linking program maturity to clinical outcomes would require a prospective study. The illustrative state-level application is synthetic and is included only to show how the modules chain together. Two systematic-review protocols (on fluoride varnish and on school sealant programs) are planned to strengthen the documented evidentiary basis for specific program parameters but have not yet been conducted, and the federal statistics reproduced here are published national estimates that should be re-verified against their primary sources, with access dates, before final deposit. The trilingual Portuguese/Spanish/English codebook was prepared using the author’s native and heritage fluency in these languages rather than a formal cross-cultural validation process (e.g., back-translation, external review, or cognitive testing), which may limit its linguistic and cultural equivalence across settings and is addressed as future work below. Finally, the 24 indicators and the scorecard weights reflect the author’s judgment in operationalizing the RE-AIM domains, informed by the literature cited above, rather than formal expert consultation, stakeholder input, or pilot testing; the weights and score bands (Tables 3-4) should accordingly be considered provisional pending empirical calibration.

Future work will address these limitations by completing the two planned systematic reviews; piloting the toolkit in one or more officially designated underserved settings to assess feasibility, inter-rater reliability of the scorecard, and the burden of equity-stratified data collection; and, over time, examining whether higher program-maturity tiers are associated with improved population-level reach and continuity. A companion program-level dashboard is envisioned to render indicator cadence and maturity trends without any patient-level view. Formal cross-cultural validation of the trilingual codebook (e.g., back-translation, external expert review, or cognitive testing with users of each language) is likewise identified as a priority line of future work.

## Conclusions

PROSPER-OH is an open, non-clinical toolkit that standardizes how community preventive oral-health programs are adapted, delivered, and, above all, measured in underserved settings. By operationalizing CFIR and RE-AIM into a 24-indicator Core Indicator Set with mandatory equity stratification, a trilingual codebook, a 0-100 Program Quality Scorecard, and an author-independent replication package aligned to federal program metrics, it offers a measurement, planning, and implementation architecture designed to facilitate standardized and potentially replicable adoption by a wide range of public-health entities without the original developer present, through its context-readiness assessment (M1) and grant-aligned reporting template (M7). Its feasibility, inter-rater reliability, cultural applicability, and relationship to program and population outcomes remain to be evaluated. It is released openly under a Creative Commons Attribution 4.0 International license for replication and feedback and has not yet been empirically validated; independent replication and critique are explicitly invited.
